# Ultrasound Microvascular Blood Flow Evaluation: A New Tool for the Management of Thyroid Nodule?

**DOI:** 10.1155/2019/7874890

**Published:** 2019-03-25

**Authors:** Carlo Cappelli, Ilenia Pirola, Elena Gandossi, Fiorella Marini, Alessandra Cristiano, Claudio Casella, Davide Lombardi, Barbara Agosti, Alberto Ferlin, Maurizio Castellano

**Affiliations:** ^1^Department of Clinical and Experimental Sciences, SSD Medicina ad indirizzo Endocrino-metabolico, University of Brescia, ASST Spedali Civili di Brescia, 25123 Brescia, Italy; ^2^Department of Molecular and Translational Medicine, 3rd Division of General Surgery, University of Brescia, ASST Spedali Civili di Brescia, 25123 Brescia, Italy; ^3^Department of Otorhinolaryngology, ASST Spedali Civili di Brescia, 25123 Brescia, Italy

## Abstract

**Background:**

Toshiba Medical System has developed a new Doppler technique [Superb Microvascular Imaging (SMI)] that has improved microvascular flow imaging. SMI depicts perinodular and intranodular thyroid microvascular flow in higher detail compared to standard colour Doppler (CD) and power Doppler (PD) imaging.

**Objective:**

Assess the nodular microvascular architecture by SMI compared to CD and PD features in a series of thyroid nodules submitted to fine needle aspiration cytology, in order to evaluate the potential of SMI in detecting thyroid cancer.

**Materials and Methods:**

From April 2016 to July 2017, 254 patients with thyroid nodules, evaluated as at high risk for malignancy in agreement with AACE/ACE/AME guidelines, were submitted to cytology. All nodules were previously submitted to ultrasound grayscale, CD, PD, and SMI evaluation. Benign and malignant nodules were stratified in accordance to the number of vessels visualised by SMI: score 1 with a maximum of two blood vessels and score 2 with three or more vessels.

**Results:**

Score 1 was found in 59.6% of benign nodules and in 17.9% of malignant nodules, whereas score 2 was found in 40.4% and in 82.1%, respectively (sensitivity 81.7%; specificity 60.5%, *p* < 0.001). Variables significantly associated with malignancy in the univariate analysis were gender (OR, 0.18; 95% CI, 0.08-0.37; *p* < 0.001), vascularity (OR, 1.91; 95% CI, 1.65-3.89; *p* < 0.001), and SMI (OR, 6.72; 95% CI, 3.89-11.59; *p* < 0.001); multivariate logistic model confirmed SMI score 2 as an independent risk factor for malignancy (OR, 6.99; 95% CI, 3.46-12.09; *p* < 0.001).

**Conclusions:**

This prospective pilot study showed that SMI can depict intranodular flow in higher detail compared to CDI and PDI, thus improving thyroid cancer detection.

## 1. Introduction

Thyroid nodularity is extremely common in clinical practice. In a large population study, clinically apparent thyroid nodules were present in 6.4 percent of women and 1.5 percent of men [1]. In surveys of unselected subjects using ultrasonography, 20 to 76 percent of women had at least one thyroid nodule [2, 3]. In Italy, an area of relative iodine deficiency, the ultrasound screening finds thyroid nodules in approximately 50% of all adults [4], and the figure climbs as high as 68% in certain regions [5]. Despite the number of thyroid nodules being impressive, the prevalence of thyroid cancer is low, amounting to about 7%-15% of cases depending on age, sex, radiation exposure history, family history, and other factors [6]. Fine-needle aspiration cytology (FNAC) is considered the most reliable test for the diagnosis of thyroid nodules, but it is not cost-effective to submit all the lesions to FNAC; therefore, ultrasound (US) evaluation has become crucial to determine when a nodule requires FNAC or sonographic follow-up. For this reason, several societal guidelines have been written to aid practitioners in making appropriate recommendations, which have also identified certain US features that suggest a higher likelihood of malignancy of a thyroid nodule [7–11]. The hypoechogenicity of the nodule, irregular margins, presence of microcalcifications, absence of halo, and a “taller than wide” shape are considered the features that are more suggestive of cancer. However, none of these are sufficiently specific to classify a lesion as malignant [12, 13].

The use of colour Doppler (CD) and power Doppler (PD) imaging for characterisation of thyroid nodule vascularity is widely used currently. It is considered a nonspecific feature for malignancy even if the presence of intranodular flow raises more concerns than if there is no flow or just perinodular flow is seen. Indeed, different studies showed conflicting data about intranodular vascularity as a sonographic risk factor for thyroid cancer. Perhaps, it depends on the fact that there is not good enough technology to detect it. However, this point remains a matter of debate among specialists worldwide. In this regard, for example, the American Thyroid Association guidelines do not include vascularity in ultrasonographic risk stratification, whereas for the AACE/ACE/AME task force it represents a high-risk feature for malignancy [8–11].

Very recently, a company (Toshiba Medical System) has developed a new Doppler technique (Superb Microvascular Imaging (SMI)) aimed at improving microvascular flow imaging through a new adaptive algorithm which removes clutter dramatically while maintaining very high frame rates.

The purpose of this pilot study was to assess the nodular microvascular architecture by means of SMI compared to standard CD and PD in a prospective series of thyroid nodules submitted to FNAC, in order to evaluate the potential of SMI in detecting thyroid cancer.

## 2. Materials and Methods

From April 2016 to July 2017, 254 patients with thyroid nodules were submitted to FNAC at our Department. All the nodules were previously evaluated as high-risk for malignancy in agreement with AACE/ACE/AME guidelines [11]. All subjects were submitted to ultrasound examination consisting of grayscale-US, CD, and PD (frame rate 10-15 Hz) followed by SMI (frame rate > 50 Hz) using an Aplio 500 ultrasound system (Toshiba Medical System, Japan) with a broad bandwidth linear array transducer (imaging frequency: 14 MHz) before being submitted to FNAC. Grayscale ultrasound was first performed to scan the nodule; subsequently, CD, PD, and SMI were performed to evaluate the vascularity both in and around the lesions. The signal was considered a real blood flow signal only if the pulsed Doppler showed an arterial or venous flow pattern. All ultrasonographic examinations were performed and recorded by the same investigator, who had >15 years of experience in thyroid ultrasound.

All the FNAC were performed using 25-gauge needles and capillary action, using a freehand technique and three passes were made/nodule. After insertion into the nodule, the needle was moved back and forth several times with a rapid but gentle stabbing motion. Cytological specimens were smeared according to the Papanicolaou technique and evaluated by experienced cytopathologists blinded to the ultrasound and Doppler results. When the smear was inadequate, FNAC was repeated once; only technically satisfactory results were considered. All the cytopathology analyses were blinded to the ultrasound and Doppler results.

Initial cytological results from the thyroid FNAC procedures were classified into five diagnostic categories based on the Italian thyroid classification system: TIR1 nondiagnostic, TIR1c nondiagnostic cystic; TIR2 nonmalignant/benign; TIR3A low-risk indeterminate lesion; TIR3B high-risk indeterminate lesion; TIR4 suspicious of malignancy; and TIR5 malignant [14]. All patients with high-risk indeterminate lesion (TIR3B) and suspicious or malignant cytology (TIR4/5) underwent surgery.

Taking into account the low false-negative rate reported in literature [8, 11], negative FNAC was considered sufficient to define the nodules as benign for statistical purposes.

The study was conducted according to the principles of the Helsinki Declaration and the guidelines of the Institutional Ethical Committee. Written informed consent was obtained from all participants.

The study was approved by the Local Ethical Committee (no. 3192).

## 3. Results

After a second round of FNAC, 18 (7.1%) patients still had inadequate cytological specimens and were excluded from further analysis. Therefore, a total of 242 nodules with valid cytological specimens were obtained from 236 patients (186 females, 50 males). A single nodule was examined in 228 patients, whereas two were biopsied in 7 patients. Nodule size (as evaluated by ultrasound) ranged from 8 to 54 mm (mean ± SD 13.3 ± 9.0 mm).

FNAC showed indeterminate lesion (TIR3B) and suspicious or malignant (TIR4/5) results in 157 nodules (65%); all underwent surgery. A diagnosis of carcinoma was histologically confirmed in 128 (81.5%).

**Figure 1:**
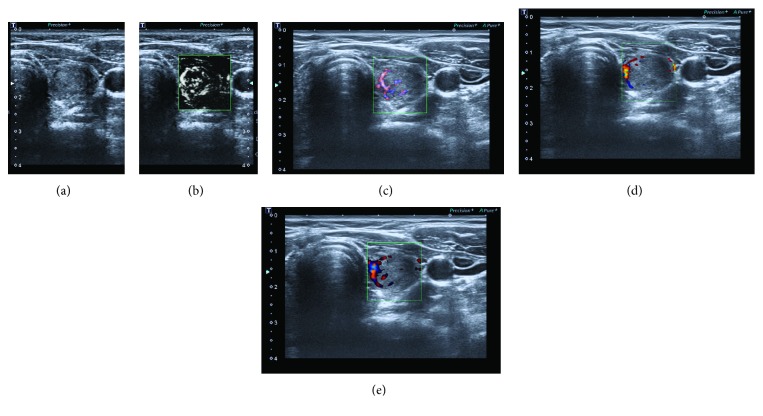
Solid nodule in the left lobe: images acquired with the five modes: grayscale US (a), monochrome (b) and colour (c) SMI, CD (d), and PD (e). Monochrome and colour SMI showed microvascular flow in greater detail compared to CD or PD.

Examples of the images acquired with the five modes (grayscale US, CD, PD, colour, and monochrome SMI) are reported in Figure 1. Monochrome and colour SMI depicted microvascular flow in greater detail compared to both CD and PD (Figure 1).

The number of blood vessels observed inside benign and malignant nodules by SMI is reported in Table 1.

**Figure 2:**
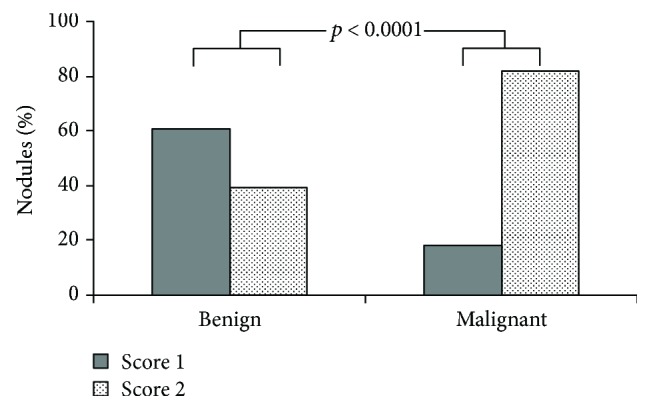
Analysis of all 242 nodules with valid cytological specimens: stratification of benign and malignant nodules in accordance to the number of vessels visualised by SMI. Score 1: maximum two blood vessels. Score 2: three or more vessels.

Benign and malignant nodules were stratified in accordance to the number of vessels visualised by SMI: score 1 with a maximum of two blood vessels and score 2 with three or more vessels. Demographic and ultrasound characteristics between patients of SMI score 1 and score 2 cohorts were superimposable except for nodular vascularity (Table 2). Following stratification, a significant different behaviour emerged in the two groups (Figure 2). In detail, score 1 was found in 59.6% of benign nodules and in 17.9% of malignancies, whereas score 2 was found in 40.4% of benign nodules and in 82.1% of malignant ones (sensitivity 81.7%, specificity 60.5%, positive predictive value 76.3%, negative predictive value 68.1%, and accuracy 73.4%; *p* < 0.001).

The 128 malignant diagnoses included 116 papillary carcinomas, 9 follicular carcinomas, and 3 Hurtle carcinomas.

There was no significant difference in age, size, hypoechogenicity, microcalcification, and blurred margins between patients with benign and malignant nodules (Table 3). Variables significantly associated with malignancy in the univariate analysis were gender (OR, 0.18; 95% CI, 0.08-0.37; *p* < 0.001), vascularity (OR, 1.91; 95% CI, 1.65-3.89; *p* < 0.001), and SMI (OR, 6.72; 95% CI, 3.89-11.59; *p* < 0.001); multivariate logistic model confirmed SMI score 2 as an independent risk factor for malignancy (OR, 6.99; 95% CI, 3.46-12.09; *p* < 0.001) (Table 4).

## 4. Discussion

The present prospective pilot study showed that SMI can depict intranodular flow in greater detail compared to CDI and PDI, thus improving thyroid cancer detection, in particular in thyroid lesion, without the need for CD and PD vascularity evaluation.

After the first report by Fujimoto et al., which described structural alteration within the thyroid gland by ultrasound evaluation in 1967 [15], this technique has become more and more established, now representing an indispensable tool in the clinical practice for thyroid evaluation. In addition, the most relevant recent guidelines and the scientific societies clearly indicate that thyroid ultrasound should be used to identify nodules with a low risk of cancer for which biopsy could be deferred [8, 10, 11]. However, none of the usual grayscale ultrasound features suggesting malignancy (hypoechogenicity, blurred margins, microcalcifications, and a “taller than wide” shape) is able to detect cancer with significant specificity and sensibility when used alone [13]. Even if vascularity is an important feature of ultrasound evaluation, no agreement has been reached regarding its assessment in thyroid nodule management. Some authors, in fact, claim that intranodular vascularity is useful for predicting thyroid malignancy [16, 17], whereas others demonstrate that this ultrasound aspect does not help to predict thyroid cancer [7, 18–20]. In this view, guidelines of different societies seem to conflict; e.g., the ATA guidelines suggest that intranodular vascularity may be reflective of follicular thyroid cancer but not of papillary cancer, and therefore they do not include it in sonographic risk factors for malignancy [8]. On the contrary, the AACE/ACE/AME guidelines include intranodular vascularity in the list of high-risk features [11]. One of the reasons of disagreement could be the difficulty to obtain the full real vascular information from both CD and PD images. In fact, although the latter is more sensitive than the former in detecting small vessels, they are both limited by clutter and overflow when used under a lower-scale condition, because the clutter (tissue motion artefacts) would overlie real low-velocity blood flow, and the overflow (blood flow signals over the lumen) would deform vessels. The recent introduction of the new Doppler technique (SMI) has improved microvascular flow imaging [21]. This has been in fact recently demonstrated in different fields, such as in breast lesions [22–24], testicular [25] hepatic tumours [26], as well as in low-velocity venous flow following pancreas transplantation [27].

In particular in breast cancer, SMI showed superiority to both colour and power Doppler imaging in detecting tumour vessels, in visualising the details of vessel morphology and both peripheral and central vascular distribution [28]. Its superiority in detecting microvascular flow imaging has also been demonstrated in another set of patients, particularly in little children. In these patients, SMI evidenced more detailed data about testicular vascular structure compared with the CD technique [25].

To date, only a few reports have been published about SMI in thyroid diseases, but they are all in line with the previous data. In particular, Flemming and colleagues showed that SMI can depict peri- and intranodular thyroid microvascular flow in greater detail compared to CD and PD in thyroid nodules [29]. This data was confirmed by Machado et al. two years later [21]. Moreover, Kong et al. have successfully demonstrated that intranodular vascularity on SMI was useful for detecting thyroid cancer in a series of 113 nodules [30]. This present study confirms and extends the previous ones. In particular, SMI increased the sensitivity for detecting thyroid cancer, showing at the multivariate logistic analysis level that the presence of three or more vessels (score 2) is significantly associated to malignancy (OR, 6.99; 95% CI, 3.46-12.09, *p* < 0.001).

Despite that intranodular vascularity as an ultrasound feature predictive of malignancy is still a matter of debate, it is well known that the recruitment of new capillary blood vessels is a tool for any tumour in growing [31].

These new vessels are so intensive and tiny at first that colour and power Doppler imaging cannot clearly show the branching details. Power Doppler imaging is limited not only by clutter but also by overflow when displaying minute vessels, which is why penetrating vessels are mistaken as peripheral vessels. On the contrary, since at lower scales there is almost no angle dependence, clutter, or overflow, SMI shows much more complete and genuine vascular branches. Therefore, many avascular nodules can currently be categorised in vascular groups, and the classification of many penetrating vessels previously mistaken as peripheral flow can be corrected.

This study has some limitations. First is selection bias of nodules: we have in fact evaluated only nodules at high risk of malignancy in accordance to AACE/ACE/AME guidelines. An unselected and larger series of nodules should be verified in the future. Second, the morphologic characteristics of vessels, such as smooth versus tortuous, were not evaluated; we only investigated the vascular pattern. Third, we correlated SMI findings with cytological results and not with histological results. Considering the low false-negative rate of FNAC reported in benign nodules [8, 11], we believe that our results can be considered reliable. However, future studies comparing SMI with histological specimens are needed.

In conclusion, the presence of three or more vessels on SMI may be a useful sign to help identify thyroid carcinoma.

## Figures and Tables

**Table 1:** Number of blood vessels observed inside benign and malignant nodules by SMI.

N. of vessels inside the nodule	Benign *n* = 114 (%)	Malignant *n* = 128 (%)
0	24 (21.2)	3 (2.3)
1-2	44 (38.6)	20 (15.6)
3-4	23 (20.1)	47 (36.7)
>5	23 (20.1)	58 (45.4)

**Table 2:** Demographic and ultrasound characteristics between patients of SMI score 1 and score 2 cohorts.

	SMI		*p* value
	SCORE 1 (*n* = 92)	SCORE 2 (*n* = 150)	
Gender (F/M)	68/23	118/27	0.127
Age (yr)	53.7 ± 12.1	54.7 ± 12.0	0.508
Nodule size (mm)	13.2 ± 11	13.3 ± 0.9	0.938
Ultrasound feature of nodule			
Hypoechogenicity (Y/N)	68/24	107/43	0.774
Microcalcification (Y/N)	38/54	65/85	0.431
Blurred margins (Y/N)	34/58	45/105	0.164
Intranodular vascularization (Y/N)	29/63	108/42	0.001

**Table 3:** Demographic and ultrasound characteristics between patients with benign and malignant nodule.

	Benign nodule	Malignant nodule	*p* value
Gender (F/M)	99/15	93/35	0.005
Age (yr)	54.7 ± 11.4	54.0 ± 12.5	0.641
Nodule size (mm)	13.1 ± 10	13.2 ± 11	0.941
Ultrasound feature of nodule			
Hypoechogenicity (Y/N)	83/31	92/36	0.873
Microcalcification (Y/N)	40/74	63/65	0.018
Blurred margins (Y/N)	37/77	52/76	0.532
Intranodular vascularization (Y/N)	48/66	89/39	0.001
SMI (score 1/2)	45/69	23/105	0.001

**Table 4:** Multivariate analysis of characteristics predicting the malignancy.

Predictors	OR (95% CI)	*p* value
Gender	0.11 (0.04-0.28)	0.001
Age	0.99 (0.96-1.01)	0.547
Hypoechogenicity	0.79 (0.56-1.13)	0.208
Microcalcification	1.34 (0.69-2.60)	0.373
Blurred margins	0.92 (0.45-1.87)	0.547
Intranodular vascularization	1.90 (1,00-3.63)	0.061
SMI	6.99 (3.46-12.09)	0.001

## Data Availability

The clinical and ultrasound data used to support the findings of this study are available from the corresponding author upon request.
